# Calibration of Discrete Element Method Parameters for a High-Fidelity Lunar Regolith Simulant Considering the Effects of Realistic Particle Shape

**DOI:** 10.3390/ma17194789

**Published:** 2024-09-29

**Authors:** Ningxi Zhou, Jian Chen, Ning Tian, Kaiwei Tian, Juehao Huang, Peng Wu

**Affiliations:** 1 State Key Laboratory of Geomechanics and Geotechnical Engineering, Institute of Rock and Soil Mechanics, Chinese Academy of Sciences, Wuhan 430071, China; zhouningxi20@mails.ucas.ac.cn (N.Z.); tianning19@mails.ucas.ac.cn (N.T.); tiankaiwei96@gmail.com (K.T.); jhhuang@whrsm.ac.cn (J.H.); wupeng21@mails.ucas.ac.cn (P.W.); 2 University of Chinese Academy of Sciences, Beijing 100049, China; 3 Hubei Key Laboratory of Geo-Environmental Engineering, Wuhan 430071, China; 4 China-Pakistan Joint Research Center on Earth Sciences, Islamabad 45320, Pakistan

**Keywords:** lunar regolith simulant, discrete element method, parameter calibration, particle shape, angle of repose

## Abstract

The Discrete Element Method (DEM) is an important tool for investigating the geotechnical properties of lunar regolith. The accuracy of DEM simulations largely depends on precise particle modeling and the appropriate selection of mesoscopic parameters. To enhance the reliability and accuracy of the DEM in lunar regolith studies, this paper utilized the high-fidelity IRSM-1 lunar regolith simulant to construct a DEM model with realistic particle shapes and conducted an angle of repose (AoR) simulation test. The optimal DEM parameters were calibrated using a combination of the Plackett–Burman test, steepest ascent test, and Box–Behnken design. The results indicate that the sliding friction coefficient, rolling friction coefficient, and surface energy significantly influence the simulation AoR. By optimizing against the measured AoR using a second-order regression model, the optimal parameter values were determined to be 0.633, 0.401, and 0.2, respectively. Under these optimal parameters, the error between the simulation and experimental AoR was 2.1%. Finally, the calibrated mesoscopic parameters were validated through a lifting cylinder test, showing an error of 6.3% between the simulation and experimental results. The high similarity in the shape of the AoR further confirms the accuracy and reliability of the parameter calibration method. This study provides a valuable reference for future DEM-based research on the mechanical and engineering properties of lunar regolith.

## 1. Introduction

As Earth’s only natural satellite, the Moon possesses a unique geographical position and abundant material resources [1,2,3], making it the primary target for space exploration and the development of extraterrestrial resources. Since the beginning of the 21st century, countries and organizations such as China, the United States, Russia, the European Space Agency, and Japan have launched a series of lunar exploration missions [4,5], primarily aimed at in situ resource utilization (ISRU) and the establishment of an International Lunar Research Station (ILRS). Lunar regolith is the primary research object and carrier of these explorations, and its unique characteristics, such as varying particle sizes and irregular shapes, present unprecedented challenges for construction in a low-gravity environment. A systematic and in-depth study of its physical and mechanical properties, such as compressibility, shear strength, and bearing capacity, is indispensable for the successful design and construction of lunar infrastructure. Understanding these properties is crucial for ensuring the safety and stability of any structures built on the Moon, whether they be for human habitation, resource extraction, or scientific research. Moreover, as lunar regolith is the primary material available for excavation and construction, understanding its behavior under lunar conditions will allow for efficient resource utilization, excavation techniques, and the development of suitable foundations for lunar bases and other structures. Research on the geotechnical properties of lunar regolith is not only crucial for the safe construction of long-term lunar habitats but also lays the foundation for expanding extraterrestrial construction to other celestial bodies.

Lunar regolith is formed through the combined effects of meteorite impacts, solar wind particle bombardment, and the lunar environment [6], resulting in complex particle morphology and characteristics that distinguish its physical and mechanical properties significantly from those of Earth’s soil. To date, the Apollo missions have returned 382 kg of lunar samples, including 115 kg of lunar regolith; the Luna missions have brought back 321 g; and the Chang’e 5 missions have collectively returned 1.73 kg of lunar regolith [7]. Due to the scarcity and value of these samples, their quantity is insufficient to meet the needs of geotechnical testing. As a result, scholars worldwide have turned to lunar regolith simulants with similar properties for related experimental research. To meet various research demands, several dozen simulants have been developed [8,9], including MLS-1 [10], JSC-1 [11], and GRC-1 [12] from the United States; CAS-1 [13], TJ-1 [14], QH-E [15], and CUMT-1 [7] from China; and KLS-1 [16], EAC-1A [17], and UoM-B [18] from other countries. Through laboratory studies using these simulants, researchers have gained a deeper understanding of the mechanical properties of lunar regolith and its interaction mechanisms with structures.

On the other hand, simulating the Moon’s unique environment, marked by low gravity, high vacuum, and extreme temperature variations, poses significant challenges on Earth. As a result, the DEM has become an effective and feasible approach for studying the mechanical properties of lunar regolith through numerical simulations, which focuses on particle properties and interactions [19,20,21,22]. Li et al. [23] and Wu et al. [24] studied the macroscopic and microscopic mechanical behaviors of lunar regolith simulant under different stress-loading paths. Hasan et al. [25] examined the strength characteristics of JSC-1A under varying confining pressures and densities. Jiang et al. [26,27] incorporated rolling resistance into the contact model to account for the shape effects of lunar regolith particles. Katagiri et al. [28] used *μ*-CT scanning to obtain the three-dimensional shapes of 74 lunar regolith particles and, combined with the DEM, studied how particle shape influences the AoR and simple shear behavior. Khademian et al. [29] investigated the effect of four specific particle shapes on the AoR under different gravity conditions.

However, despite significant progress having been made in studying the physical and mechanical properties of lunar regolith and its simulants, numerical simulations based on the DEM still have some limitations. One issue is the simplification of complex lunar regolith particles into idealized spherical particles in many studies, which is due to the challenges in simulating real particle shapes and the constraints of computational capacity. Such simplifications often overlook the significant impact of particle shape on physical and mechanical properties [30,31,32]. Additionally, the selection of appropriate mesoscopic parameters in the DEM is crucial for ensuring the accuracy and reliability of simulation results [33,34]. Commonly used parameter calibration methods, such as the ‘trial and error’ approach, are often time-consuming and inefficient, particularly when dealing with models that contain many unknown parameters. The iterative nature of trial and error can lead to excessive computational demands, requiring multiple simulations or tests before arriving at satisfactory parameter values. This not only prolongs the calibration process but also limits the practical applicability of these methods in large-scale or complex models. On the other hand, machine learning-based parameter calibration techniques, while promising, present their own set of challenges. These methods typically require a large dataset of computational samples to train the model effectively, and the complexity of developing and fine-tuning the machine learning algorithms adds further barriers. The intricate nature of machine learning models can also obscure the physical relationships between parameters, making them less interpretable for practical engineering applications. Additionally, the computational resources required to generate sufficient training data can be prohibitive, especially for high-fidelity simulations [35,36]. To overcome these limitations, many researchers have turned to experimental design methods, such as response surface methodology (RSM) [37,38,39]. RSM offers a more efficient and systematic way of exploring the parameter space, allowing researchers to optimize model parameters with a minimal number of experiments or simulations. By building a predictive model of the relationship between the parameters and the desired outputs, RSM enables a structured approach to parameter calibration, leading to faster convergence on optimal values. This significantly improves the efficiency of the calibration process, reducing computational costs while maintaining accuracy. However, although RSM has been applied in various studies to calibrate soils and rock materials, the application of this method in calibrating the mesoscopic parameters of lunar regolith particles remains relatively limited.

In this study, a newly developed high-fidelity lunar regolith simulant named IRSM-1 was firstly introduced, demonstrating its similarity to real lunar regolith in terms of chemical and mineral composition, as well as physical and mechanical properties. Image processing techniques were then used to capture and analyze the realistic shapes of 1250 IRSM-1 particles, and a corresponding discrete element model was created using a bubble packing algorithm. Based on physical experiments, AoR simulation considering the realistic shapes of IRSM-1 particles was conducted, and the DEM mesoscopic parameters were calibrated using response surface methodology. Finally, the effectiveness and accuracy of the calibrated parameters were further validated through a lifting cylinder test. This study provides valuable reference and support for research on the mechanical and engineering properties of lunar regolith using the DEM.

## 2. Materials and Methods

### 2.1. IRSM-1 Lunar Regolith Simulant

**Figure 1:**
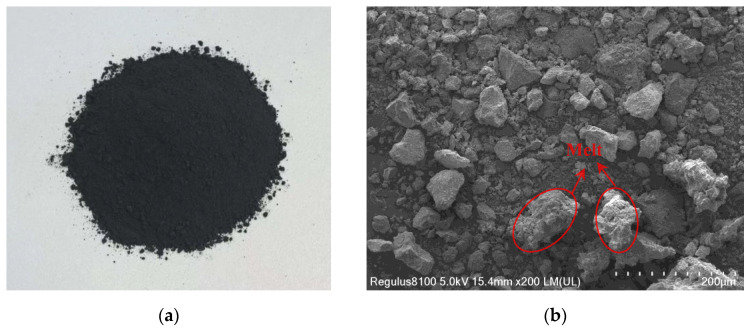
Appearance (**a**) and micro morphology (**b**) of the IRSM-1 lunar regolith.

The IRSM-1 lunar regolith simulant [40] is made from basaltic volcanic ash and ilmenite, processed through high-temperature sintering, impact crushing, and sieving. It is classified as a high-titanium simulant, with its appearance shown in Figure 1a. Figure 1b displays the micro morphology of IRSM-1 particles captured by scanning electron microscopy (SEM). As shown in the image, IRSM-1 particles exhibit complex shapes, including elongated and angular particles, as well as irregular particles with bumps and notches. These irregular shapes stem from the partial melting of raw materials during the high-temperature sintering process.

**Figure 2:**
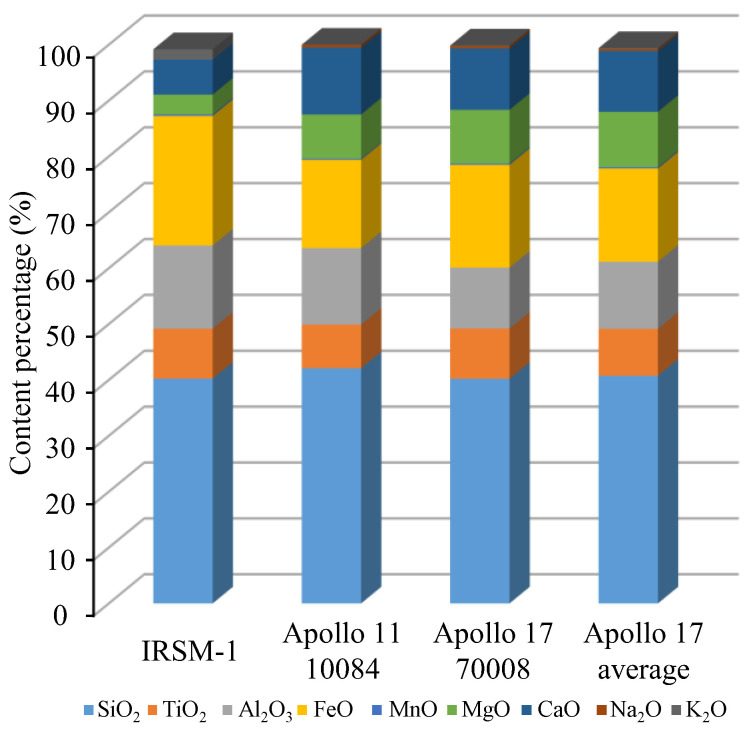
Comparison of the chemical composition of IRSM-1 with Apollo lunar regolith samples.

**Figure 3:**
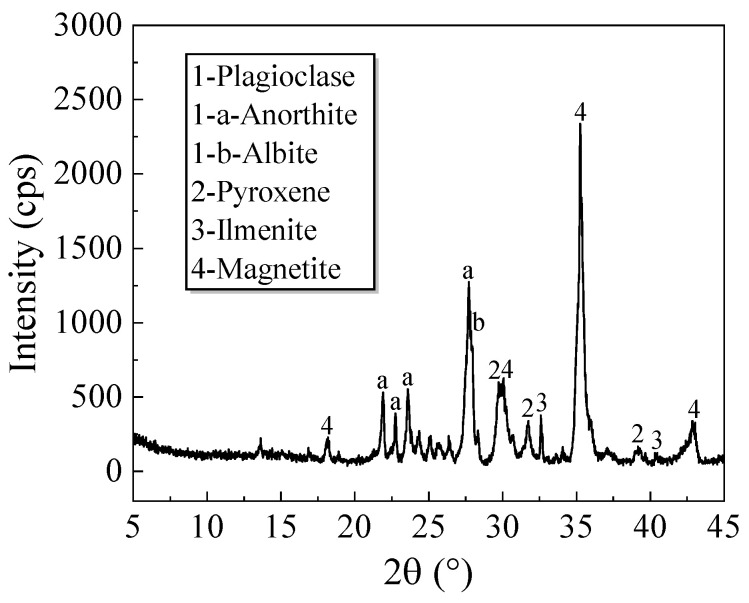
Mineralogical analysis of IRSM-1 lunar regolith.

The chemical composition and mineral content of IRSM-1 were analyzed using X-ray Fluorescence Spectrometry (XRF) and X-ray Diffraction (XRD), respectively, as shown in Figure 2 and Figure 3. Figure 2 reveals that the primary oxides in IRSM-1 include SiO_2_, TiO_2_, Al_2_O_3_, and FeO, along with smaller amounts of MgO, CaO, and K_2_O. When compared with the chemical composition of lunar samples collected during the Apollo 11 and 17 missions [6,8], it is evident that the chemical composition of IRSM-1 closely resembles that of the Apollo lunar samples. Figure 3 indicates that IRSM-1 is primarily composed of minerals such as plagioclase, pyroxene, ilmenite, and magnetite, which are similar to the mineral composition of the Apollo 17 lunar samples.

**Figure 4:**
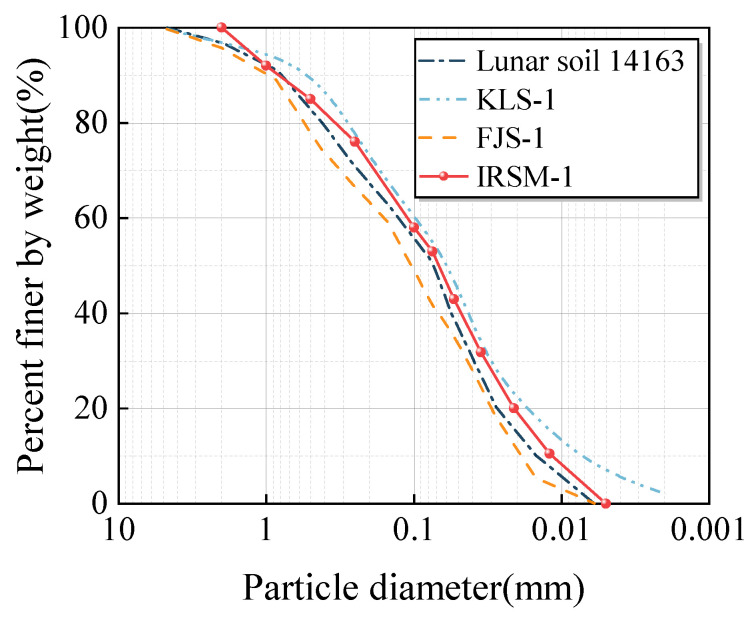
Comparison of the grain size distribution curves between IRSM-1 and other samples.

In terms of physical and mechanical properties, the relative density of IRSM-1 is 3.26, while the relative density of lunar samples ranges from 2.9 to 3.4 [6]. Figure 4 compares the grain size distribution curves of lunar sample 14163 with those of the IRSM-1, KLS-1, and FJS-1 simulants [16]. Triaxial compression tests were conducted on IRSM-1 at different relative densities, and Mohr–Coulomb strength criteria were employed to calculate the cohesion and internal friction angle of IRSM-1, which were determined to be 0.81–1.63 kPa and 44.1°–52.8°, respectively, as shown in Table 1. Additionally, Table 1 lists the shear strength data for lunar samples and several simulants, indicating that the cohesion and internal friction angle of IRSM-1 fall within the ranges of lunar samples. This suggests that IRSM-1 closely resembles real lunar regolith in terms of its geotechnical properties.

By analyzing the particle morphology, gradation curve, mineral composition, chemical composition, relative density, and shear strength of IRSM-1, it is evident that IRSM-1 effectively replicates the physical and mechanical properties of real lunar regolith, as well as its complex particle morphology. This makes IRSM-1 a high-fidelity simulant, suitable for studies on lunar regolith properties.

#### 2.1.1. Image Processing

**Figure 5:**
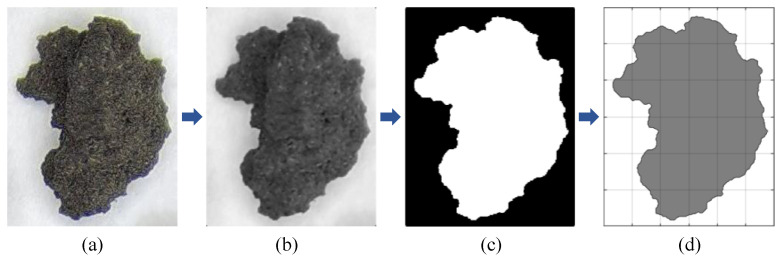
Contour coordinate extraction process of IRSM-1 particle: (**a**) Import the image. (**b**) Grayscale processing. (**c**) Threshold segmentation. (**d**) Contour extraction.

To analyze the morphological characteristics of IRSM-1 particles and to provide templates of actual particle shapes for subsequent DEM simulations, this study utilized microscopy and image processing techniques to obtain contour data of IRSM-1 particles. The specific procedure is shown in Figure 5. The detailed steps are as follows: First, high-definition digital images of the simulant particles were captured using an industrial microscope and imported into MATLAB, as shown in Figure 5a. Next, the imported color images were converted to grayscale, and a median filtering algorithm was applied to remove noise and enhance the images. Then, the grayscale images were binarized, and the holes within the particles were filled, as shown in Figure 5c. Finally, the boundary between the particles and the image background was identified by searching for pixel values, and the contour coordinates of the particles were extracted.

#### 2.1.2. Particle Shape Analysis

**Figure 6:**
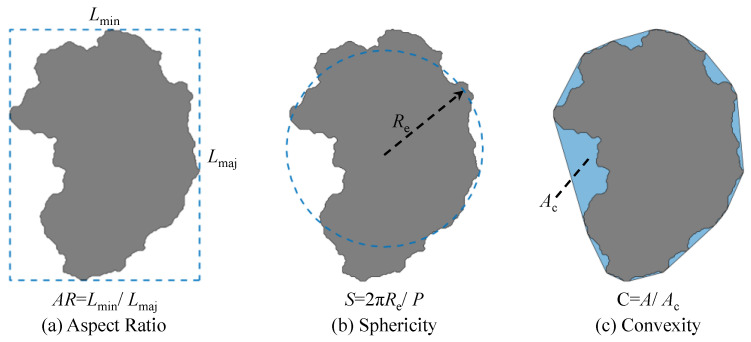
Definition and calculation of particle shape parameters.

After obtaining the contour coordinates of IRSM-1 particles, the aspect ratio (*AR*), sphericity (*S*), and concavity (*C*) were employed as morphological evaluation parameters to quantify the particle shape characteristics. The calculation methods of these parameters are shown in Figure 6. Specifically, *AR* is defined as the ratio of the particle’s minor axis length (*L*_min_) to its major axis length (*L*_maj_). *S* is defined as the ratio of the circumference of an equivalent circle with the same area as the particle (2*πR*_e_) to the actual perimeter of the particle (*P*), which describes how closely the particle’s geometry resembles an ideal circle. *C* is defined as the ratio of the particle’s area (*A*) to its convex hull area (*A*_c_).

#### 2.1.3. Angle of Repose Experiment

**Figure 7:**
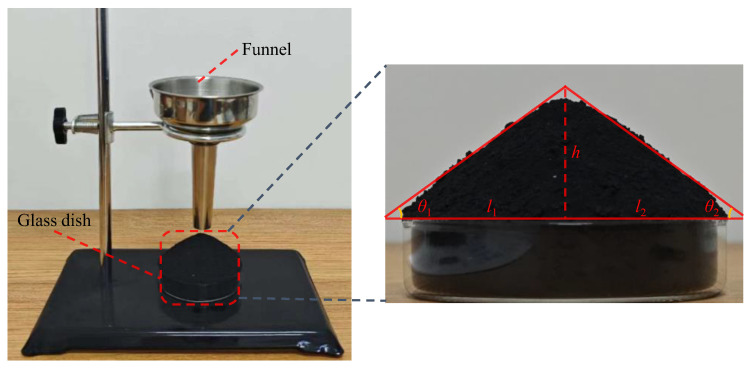
Angle of repose experiment of IRSM-1 lunar regolith simulant.

The AoR experiment is a simple and effective way for testing the friction and flow characteristics of powders or granular materials. It is also widely used for calibrating the mesoscopic parameters in the DEM. The experimental setup used in this study is shown in Figure 7. During the experiment, IRSM-1 particles were slowly poured through a funnel into a glass dish at the bottom until a stable pile angle was formed. The AoR was then captured using a camera, as shown in Figure 7, and its value was calculated according to Equation (1).

(1) $$\theta =\frac{1}{2}\cdot (\arctan\frac{h}{l_{1}}+\arctan\frac{h}{l_{2}})$$

### 2.2. The DEM Simulation

The numerical simulations in this study were conducted using the PFC 7.0 software developed by Itasca. The computational principles of the DEM can be found in reference [41]. The selection of the contact model between particles in DEM simulations is important for ensuring the accuracy and correctness of the results. Previous studies [26,42] have demonstrated that the high vacuum and low gravity on the lunar surface lead to non-negligible van der Waals forces between lunar regolith particles, resulting in a certain degree of cohesion. The Johnson–Kendall–Roberts (JKR) contact model is an extension of the Hertz–Mindlin model, incorporating an attractive force component to model cohesion and accounting for the influence of van der Waals forces between particles. Additionally, this model includes a rolling resistance mechanism, allowing it to account for the complex surface roughness of lunar regolith particles. Based on these considerations and the research experiences of Modenese [43] and Zhu [38], the Hertz–Mindlin with JKR model was selected as the contact model for IRSM-1 particles in this study.

#### 2.2.1. JKR Contact Model

As previously mentioned, the JKR model is an extension of the Hertz–Mindlin model and can simulate the cohesion caused by surface energy interactions between lunar regolith particles. A detailed description of this contact model can be found in the PFC documentation. In the JKR model, the forces involved include the contact force *F_JKR_*, damping force *F_d_*, and rolling resistance moment *M_r_*, with their calculation formulas provided below, as follows:

(2) $$F_{n}^{JKR}=\frac{4E^{*}a^{3}}{3\bar{R}}-\sqrt{16\pi \gamma E^{*}a^{3}}$$

(3) $$F_{s}^{JKR}=\mu \left(F_{n}^{JKR}+2F_{po}\right)$$

(4) $$F_{n}^{d}=2\beta_{n}\sqrt{m_{c}k_{n}^{t}}{\dot{\delta }}_{n}$$

(5) $$F_{s}^{d}=\left(-2\beta_{s}\sqrt{m_{c}k_{s}^{t}}\right){\dot{\delta }}_{s}$$

(6) $$M_{r}=\mu_{r}\bar{R}\left(F_{n}^{JKR}+2F_{po}\right)$$

where $F_{n}^{JKR}$ and $F_{s}^{JKR}$, $F_{n}^{d}$ and $F_{s}^{d}$, $k_{n}^{t}$ and $k_{s}^{t}$, $\beta_{n}$ and $\beta_{s}$, and ${\dot{\delta }}_{n}$ and ${\dot{\delta }}_{s}$ represent the normal and tangential components of the contact force, damping force, stiffness, critical damping ratio, and velocity, respectively. The term *a* denotes the contact patch radius, while *F_po_* refers to the pull-off force. The parameters *γ*, *μ*, and *μ_r_* represent the surface energy, sliding friction coefficient, and rolling friction coefficient of the particles, respectively. $m_{c}$, $E^{*}$, and $\bar{R}$ represent the effective contact mass, effective Young’s modulus, and effective contact radius, with their corresponding calculation formulas provided below, as follows:

(7) $$M_{r}=\mu_{r}\bar{R}\left(F_{n}^{JKR}+2F_{po}\right)$$

(8) $$E^{*}={\left(\frac{1-\nu_{1}}{2G_{1}}+\frac{1-\nu_{2}}{2G_{2}}\right)}^{-1}$$

(9) $$\bar{R}=\frac{R_{1}R_{2}}{R_{1}+R_{2}}$$

where *m*, *R*, *G*, and *ν* represent the mass, radius, shear modulus, and Poisson’s ratio of the contact particles, respectively.

#### 2.2.2. Particle Shape Rebuilding

After extracting the contours of IRSM-1 particles, they were imported into the PFC 7.0 software, where the bubble packing algorithm [44] was utilized to generate clumps and to represent the particle morphology. The bubble packing algorithm is a simple yet efficient particle shape-rebuilding technology that involves two control parameters: *ρ* and *φ*. Here, *ρ* represents the ratio of the smallest to largest ball, while *φ* is the circle-to-circle intersection angle at the contact between two balls. When *φ* = 0°, the two balls are externally tangent to each other, while *φ* = 180° means two balls, with one being the inscribed circle of the other one.

**Figure 8:**
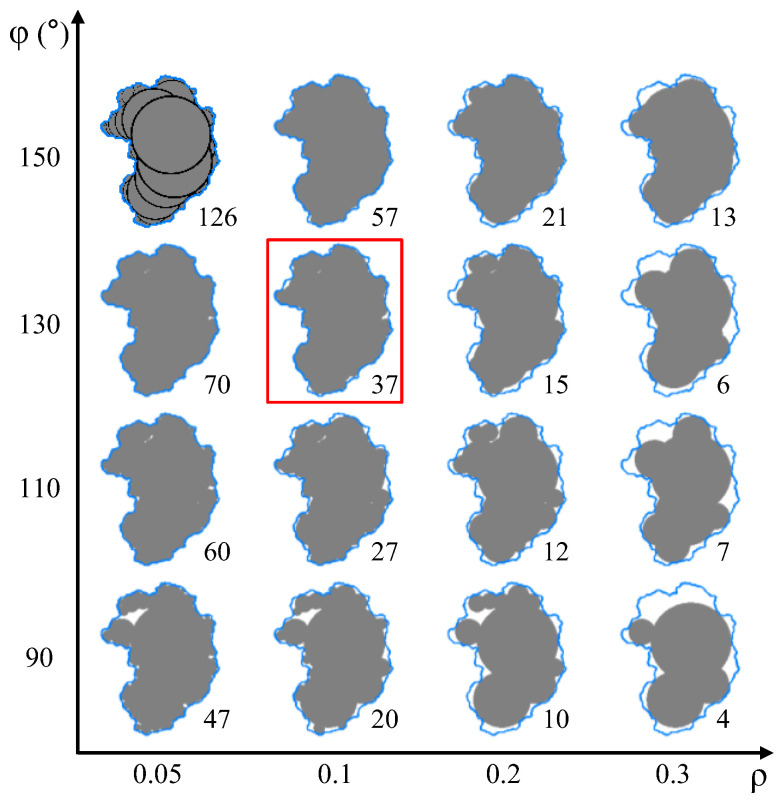
Comparison of clumps generated under different parameter combinations.

In the DEM, a clump is a rigid assembly of rigid spherical pebbles. The more pebbles used to generate a clump, the better it simulates the actual shape of the particle, but the more computational resources that are required to run an analysis involving it. The effectiveness of a clump in simulating the actual particle shapes under different parameter combinations is shown in Figure 8. It can be observed that as *ρ* decreases and *φ* increases, the number of balls included in a clump increases, thereby enhancing the accuracy of the simulated shape. Table 2 lists the area ratio of the generated clump to the actual particle for various parameter combinations. To balance simulation accuracy and computational efficiency, in this study, the *ρ* and *φ* were set to 0.1 and 130°, respectively, as indicated by the box in Figure 8. This corresponded to 37 pebbles being used to generate a clump and represent a particle with an area ratio of 0.960. Using this method, 1250 clump templates of varying shapes were produced.

#### 2.2.3. DEM Model of Repose Angle

**Figure 9:**
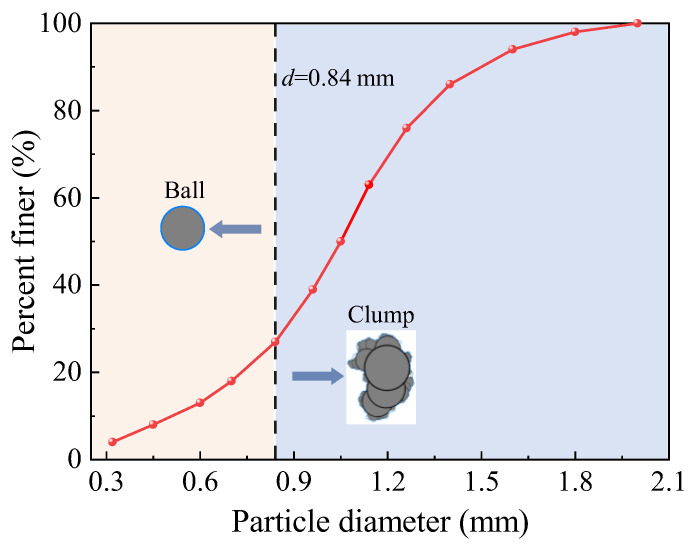
Grain size distribution curves used in DEM model.

The AoR DEM simulation was conducted following these steps. First, rigid walls were created according to the experimental setup shown in Figure 7 to simulate the funnel and glass dish. A sufficient number of particles were then generated inside and above the funnel. Given the small grain size of IRSM-1, accurately modeling the full grain size distribution in the DEM model would result in an excessive number of particles, exceeding the computational capacity of the available computer resources. Therefore, in this study, the particle size of IRSM-1 was moderately scaled up for numerical simulation, with the gradation curve used shown in Figure 9.

**Figure 10:**
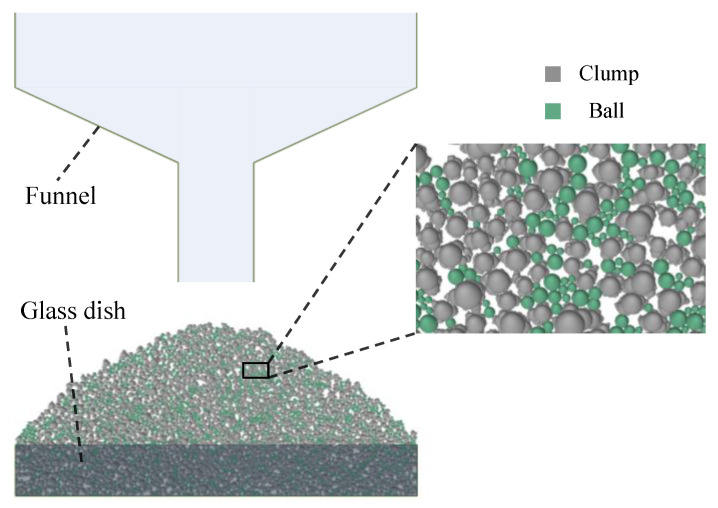
DEM simulation test of repose angle.

Next, to enhance computational efficiency while ensuring that the DEM particles retained realistic shape characteristics, only balls with a diameter greater than 0.84 mm, accounting for 73% of the total, were replaced with a clump that had actual shapes, as shown in Figure 9. The shapes of these clumps were randomly selected from the 1250 clump templates generated earlier, ensuring that the DEM particles closely resembled the morphological characteristics of the IRSM-1 simulant. Finally, gravity was applied to the model, and the simulation was initiated. The particles fell and gradually accumulated under gravity. When the ratio of the average unbalanced force to the contact force in the model fell below 1 × 10⁻⁵, the slope was considered to have reached a stable state, resulting in the final angle of repose, as shown in Figure 10.

The input DEM parameters for the model and their corresponding ranges are listed in Table 3. These parameters were used in the subsequent Plackett–Burman test.

### 2.3. The Response Surface Methodology

As shown in Table 3, the AoR simulation in this study involved eight DEM parameters. As outlined in the introduction, to efficiently identify the most significant parameters and optimize their values, a multi-step response surface methodology was employed, which included the Plackett–Burman test, steepest ascent test, and Box–Behnken design.

#### 2.3.1. Plackett–Burman Test

The Plackett–Burman (P-B) test is a statistical screening method designed to efficiently identify the most significant factors influencing a response variable. By using a fractional factorial design, the P-B test minimizes the number of experimental runs while focusing on the main effects of each factor, assuming that interactions are negligible. This makes it ideal for quickly determining which parameters have the most substantial impact on the outcome.

In this study, the P-B test was applied to screen the DEM parameters to identify those most affecting the angle of repose (AoR). The factors and levels used in the P-B test are shown in Table 3. This approach allowed us to focus on the key parameters for further optimization.

#### 2.3.2. Steepest Ascent Test

The steepest ascent test is an optimization technique used to efficiently move toward the optimal region of the response surface. It involves adjusting the levels of significant factors in the direction where the response variable changes most rapidly, based on the results from the P-B test. This method is useful after the key parameters have been identified, as it helps refine the search for optimal values without the need for an extensive number of experiments.

In this study, the steepest ascent test was used to adjust the significant DEM parameters identified in the P-B test to further improve the accuracy of the AoR simulation.

#### 2.3.3. Box Behnken Design

The Box–Behnken design (BBD) test is an efficient response surface methodology used to estimate second-order effects with fewer experimental runs. This method allows the establishment of a quadratic regression model between the three key parameters and the angle of repose. The significant parameters identified in the P-B test were employed in a three-factor, three-level response surface test, with the center points determined based on the results from the steepest ascent tests. The BBD approach efficiently captures potential interactions between the key factors, enabling a more precise optimization of the AoR.

## 3. Results and Discussion

### 3.1. Properties of IRSM-1 Simulant

#### 3.1.1. Particle Morphology Characteristics

**Figure 11:**
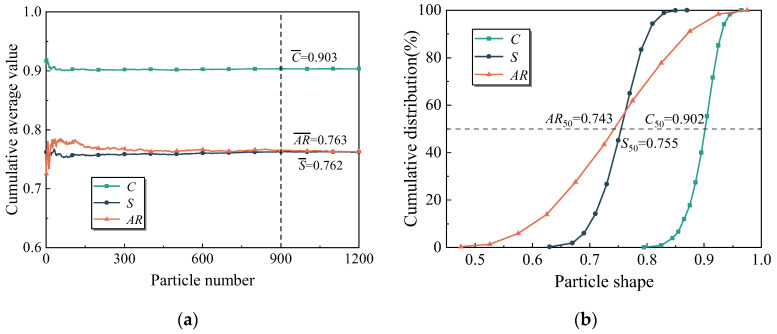
Particle shape characteristics of IRSM-1. (**a**) Cumulative average value, (**b**) cumulative distribution.

Figure 11 shows the cumulative average values and cumulative distribution curves of the aspect ratio, sphericity, and concavity for IRSM-1 particles. As shown in Figure 11a, as the number of particles analyzed increases, the cumulative average values of the shape parameters for IRSM-1 particles stabilize. After the number of particles reaches 900, the average values of *AR*, *S*, and *C* show little change. Therefore, the 1250 particles selected in this study are sufficient to represent the shape characteristics of IRSM-1 particles. Figure 11b shows that the distribution ranges for *AR*, *S*, and *C* are 0.5–0.95, 0.63–0.85, and 0.82–0.96, respectively, with median values of 0.743, 0.755, and 0.902.

#### 3.1.2. Results of Repose Angle Experiment

After capturing the stable AoR, as shown in Figure 8, the angle (*θ*) was calculated using Equation (4). The experiment was repeated five times, and the average value was taken. The final AoR of the IRSM-1 was determined to be 35.66°, which was used as the target value for optimizing the parameters in subsequent DEM simulations.

### 3.2. The Calibration Procedure of DEM Parameters

#### 3.2.1. Design and Results of P-B Test

In this study, the Design-Expert 11 software was employed to conduct the Plackett–Burman test design, with the AoR of the IRSM-1 as the response variable. Eight DEM input parameters were selected as experimental factors, and two levels—high (+1) and low (−1)—were set, as shown in Table 3. The test design included one central point, resulting in a total of 13 simulation tests. The P-B test design and results are presented in Table 4.

Table 5 presents the analysis of variance (ANOVA) results from the Plackett–Burman test, which was conducted using the Design-Expert 11 software. The table shows that the model has a *p*-value of 0.006, which is less than 0.01, and an adjusted coefficient of determination $R_{adj}^{2}=0.965$, indicating that the regression model is significant, has high credibility, and can effectively predict the trends in parameter changes. By comparing the *p*-values of the parameters, it was found that the three parameters with the greatest impact on the AoR of the IRSM-1 were, in order, the sliding friction coefficient (*μ*_1_), the rolling friction coefficient (*μ*_r1_), and surface energy (*γ*). Their *p*-values were all less than 0.01, making them highly significant. Other DEM parameters, such as the shear modulus (*G*) and Poisson’s ratio (*ν*), had a smaller influence on the AoR, with *p*-values greater than 0.05, and were therefore considered statistically insignificant. This result aligns with our expectations and previous research findings [38,45,46]. Therefore, in the subsequent steepest ascent test, these three most significant parameters were used as experimental factors to further determine their optimal ranges, while the remaining parameters were set to the average of the two levels as input values for the DEM simulation.

#### 3.2.2. Design and Results Analysis of Steepest Ascent Test

Based on the three key parameters identified in the P-B design, a steepest ascent test was designed and conducted, with the results shown in Table 6. As seen in Table 6, as the values of these three parameters increase, the relative error between the simulation and experimental AoR initially decreases and then increases. The minimum relative error, only 2.3%, occurs under the parameters used in Test 4. Therefore, the parameter values from Test 4 were selected as the central levels, with the values from Tests 3 and 5 used as the low and high levels, respectively, for the subsequent Box–Behnken response surface optimization test. The value ranges for *μ*_1_, *μ*_r1_, and *γ* for IRSM-1 particles were determined to be 0.6–0.8, 0.4–0.5, and 0.12–0.2, respectively.

#### 3.2.3. Box–Behnken Design and Regression Model

To further explore the relationship between the AoR of IRSM-1 and the parameters *μ*_1_, *μ*_r1_, and *γ*, and to determine the optimal parameter combination, a Box–Behnken response surface design was conducted using Design-Expert 11 software. The parameter value ranges obtained from the P-B test and the steepest ascent test were used as the basis for this design. A total of 15 AoR experiments were conducted, including three repeated tests at the central level. The experimental design and results are shown in Table 7.

A quadratic regression analysis and multivariate regression fitting were performed on the AoR results from Table 7, and the analysis of variance results from the Box–Behnken test are presented in Table 8. The results indicate that the quadratic regression model for the AoR is highly significant (*p* = 0.0002), with a coefficient of determination $R^{2}=0.990$ and an adjusted coefficient of determination $R_{adj}^{2}=0.973$; the lack-of-fit term is not significant (*p* = 0.074 > 0.05), indicating that the regression model has high precision. The model’s coefficient of variation is 0.59%, and the adeq precision is 24.8, which is greater than 4, suggesting that the experimental results are reliable and that the model can accurately predict the AoR of IRSM-1. Additionally, by comparing the *p*-values of the regression terms, it is evident that the parameters *μ*_1_, *μ*_r1_, and *γ* have a significant impact on the AoR (*p* < 0.01). Based on the data in Table 8, binary regression fitting was performed to establish the regression equation for the angle of repose θ with respect to the parameters *μ*_1_, *μ*_r1_, and *γ*, as follows:

(10) $$\begin{array}{c} \theta =-34.62+78.28\mu_{1}+106.05\mu_{r1}+132.94\gamma +1.7\mu_{1}\mu_{r1}-80.99\mu_{1}\gamma \\ -169.40\mu_{r1}\gamma -37.18\mu_{1}^{2}-71.85\mu_{r1}^{2}+36.91\gamma^{2} \end{array}$$

**Figure 12:**
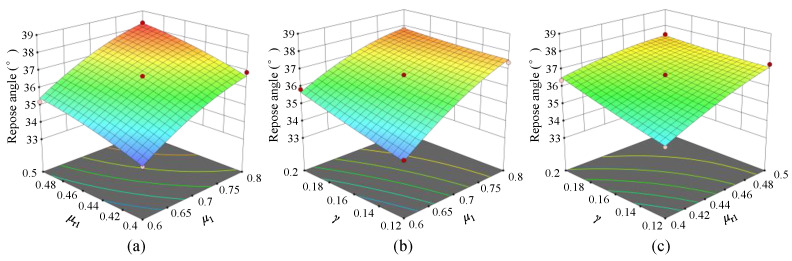
Influence of interaction terms on angle of repose: (**a**) Interaction between *μ*_1_ and *μ*_r1_. (**b**) Interaction between *μ*_1_ and *γ*. (**c**) Interaction between *μ*_r1_ and *γ*.

Figure 12 shows the response surface plots illustrating the interaction effects of various parameters on the AoR, based on the regression Equation (10). By comparing the downward trends in the response surfaces, it can be observed that the significance of the impact on the AoR follows the order *μ*_1_ > *μ*_r1_ > *γ*, which is consistent with the conclusion presented earlier.

### 3.3. Optimal DEM Parameter Determination and Validation

#### 3.3.1. Parameter Determination by the Regression Model

To determine the optimal combination of DEM parameters, the AoR obtained from the physical experiment of the IRSM-1 was used as the optimization target (θ = 35.66°). Non-significant parameters were set to the median values, and optimization was performed using Design-Expert 11 software. The objective function and constraints are shown in Equation (11).

(11) $$\{\begin{array}{c} \theta =35.66{}^{\circ}\\ 0.6\leq \mu_{1}\leq 0.8\\ 0.4\leq \mu_{r1}\leq 0.5\\ 0.12\leq \gamma \leq 0.20 \end{array}$$

After optimizing the parameters using the above equation, 100 sets of optimized solutions were obtained. These optimized solutions were then employed as DEM input parameters for the AoR simulation tests. The angle of repose results from the simulation and physical test were compared to identify the set of optimized parameters that most closely matched the value and shape of the physical AoR. The best match was found with *μ*_1_ = 0.633, *μ*_r1_ = 0.401, and *γ* = 0.2.

**Figure 13:**
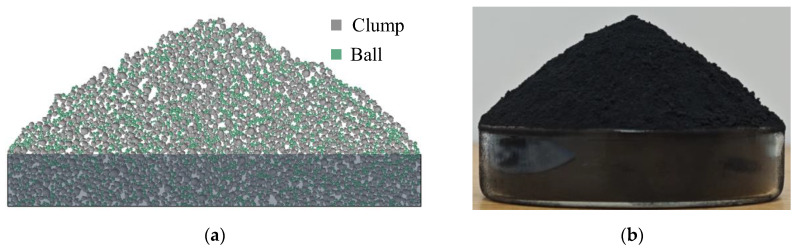
Comparison of the AoR results between simulation and physical tests: (**a**) The simulation test, (**b**) the physical test.

Figure 13 shows the comparison between the simulation and physical test results using these optimal parameters. The results demonstrated that the simulation AoR was 34.94°, with a deviation of only 2.1% from the physical test result. The high similarity in both shape and angle indicates that the selected optimal simulation parameters are accurate and effective.

#### 3.3.2. Parameter Validation by the Lifting Cylinder Test

**Figure 14:**
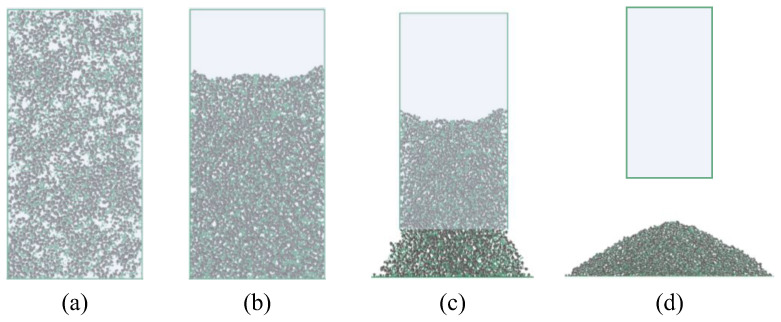
Simulation process of the lifting cylinder test: (**a**) Particle generation, (**b**) settling, (**c**) cylinder lifting, (**d**) repose angle formation.

After determining the optimal parameter combination, a lifting cylinder test was conducted to validate the effectiveness and accuracy of the method in calibrating the DEM parameters of the IRSM-1 lunar regolith simulation. The testing process is illustrated in Figure 14. First, a sufficient number of particles were generated within a cylindrical region with a porosity of 0.35, as shown in Figure 14a. The cylinder had a radius of 20 mm and a height of 80 mm. The grain size distribution and clump proportions were kept consistent with the previous AoR simulation test, as shown in Figure 9. Similarly, the DEM parameters used in the simulation were the same as those in the AoR test. The values for the three significant parameters, *μ*_1_, *μ*_r1_, and *γ*, were 0.633, 0.401, and 0.2, respectively, while the remaining parameters were set to the average of the low and high levels listed in Table 3. After generating the ball and clump particles, gravity was applied to the model, allowing the particles to gradually settle within the cylindrical region, as shown in Figure 14b. Then, the cylinder was lifted upward at a speed of 25 mm/s, allowing the particles to accumulate on the plate, forming an angle of repose, as shown in Figure 14c. Once all the particles had settled and the slope stabilized, the Aor was calculated using Equation (1).

**Figure 15:**
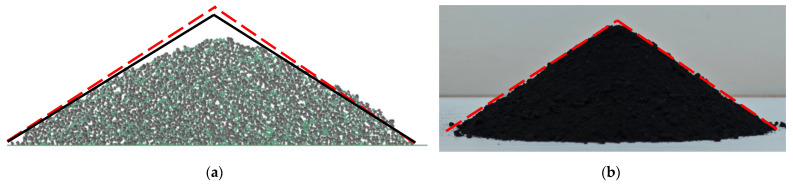
Results of the lifting cylinder test: (**a**) The simulation test, (**b**) the physical test.

Both the physical and simulation tests were repeated five times, and the average values of results were calculated. The measured AoR from the physical and simulation tests were 34.03° and 31.89°, respectively, with a 6.3% relative error. The comparison of the AoR shapes is shown in Figure 15, where the high similarity in shape and angle indicates the feasibility of this method in the parameter calibration of lunar regolith simulant particles.

## 4. Conclusions

This paper conducted a calibration of DEM parameters for the IRSM-1 lunar regolith simulant, taking into account the effects of realistic particle shapes. The response surface methodology was employed to quickly identify significant parameters and, in combination with physical experiments, determine their optimal values. Firstly, a high-fidelity lunar regolith simulant, IRSM-1, was used as the test material, and its similarity to real lunar regolith was demonstrated in terms of chemical composition, mineral composition, and physical and mechanical properties. Subsequently, image processing techniques were utilized to capture and analyze the realistic shapes of 1250 IRSM-1 particles, and corresponding discrete element models were established. Based on physical experiments, an angle of repose simulation considering the realistic shapes of IRSM-1 particles was conducted, and the DEM mesoscopic parameters were calibrated utilizing the P-B test, steepest ascent test, and Box–Behnken design. The effectiveness and accuracy of the parameter calibration method and the optimized parameters were further validated through the lifting cylinder test. The main conclusions of this study are as follows:

(1) The IRSM-1 lunar regolith simulant exhibits composition, physical, and mechanical properties that closely resemble those of real lunar regolith, making it a suitable substitute for lunar regolith research. The morphology of the IRSM-1 particles is complex, with median values of aspect ratio, sphericity, and concavity being 0.743, 0.755, and 0.902, respectively. A discrete element model was constructed for 1250 IRSM-1 particles utilizing the bubble packing algorithm, enabling the AoR simulation based on realistic IRSM-1 particle shapes.
(2) The Plackett–Burman design was employed to quickly identify the three parameters with the most significant impact on the AoR of IRSM-1 particles: sliding friction coefficient *μ*_1_, rolling resistance coefficient *μ*_r1_, and surface energy *γ*. The steepest ascent test was then employed to determine the optimal parameter ranges. Finally, a quadratic regression equation relating the AoR to these three significant parameters was developed using the Box–Behnken test. The optimal parameters were determined to be 0.633, 0.401, and 0.2, respectively, with the physical AoR (*θ* = 35.66°) as the optimization target.
(3) The simulation AoR was 34.94° using the optimal parameter combination, with a deviation of only 2.1% from the physical test result. Additionally, the lifting cylinder test was conducted, where the AoR results of the simulation and physical test were 31.89° and 34.03°, respectively, leading to an error of 6.3%. The high similarity in both angles and shapes of the AoR from the tests confirms the reliability and accuracy of the parameter calibration for the IRSM-1 lunar regolith simulant.

Overall, this preliminary study provides a quick and effective method for parameter calibration in discrete element simulations based on the realistic shapes of lunar regolith particles. Employing realistically shaped clumps instead of balls in the simulations significantly enhanced the reliability and accuracy of the numerical models, which is particularly important for particles with complex morphologies. In future DEM studies considering the shape of lunar regolith particles, the approach outlined in this paper can be utilized to quickly and accurately calibrate DEM parameters. This will facilitate the simulation of their mechanical properties and deepen our understanding of the geotechnical behavior of lunar regolith and its simulants.

## Figures and Tables

**Table 1:** Shear strength of IRSM-1 and lunar regolith simulants.

Material	Cohesion (kPa)	Friction Angle (°)
IRSM-1	0.81–1.63	44.1–52.8
Lunar regolith	0.44–3.8	41–55
TJ-1	0.86	47.6
JSC-1	6.2	49.5
FJS-1	3–8	32.5–39.4
MLS-1	0.9	37
KLS-1	1.85	44.91

Data are obtained from [7,16,40].

**Table 2:** The area ratio of clump to particle under different parameter combinations,%.

Parameters	Radius Ratio *ρ*			
	0.05	0.1	0.2	0.3
The distance *φ*: degrees				
90	94.41	90.76	86.34	75.71
110	97.22	94.13	90.05	82.53
130	97.82	96.00	90.92	81.66
150	98.47	97.12	93.52	89.70

**Table 3:** DEM parameters of Plackett–Burman test.

Symbol	DEM Parameters	Levels	
		−1	+1
*G*	Shear modulus (MPa)	40	80
*ν*	Poisson’s ratio	0.1	0.3
*μ*_1_	Sliding friction coefficient of particle	0.4	0.9
*μ*_r1_	Rolling friction coefficient of particle	0.2	0.7
*β*	Critical damping ratio	0.3	0.7
*γ*	Surface adhesion energy (J/m^2^)	0.02	0.12
*μ*_2_	Sliding friction coefficient between particle and wall	0.3	0.6
*μ*_r2_	Rolling friction coefficient between particle and wall	0.15	0.55

**Table 4:** Design and results of the Plackett–Burman test.

Number	*G*	*ν*	*μ*_1_	*μ*_r1_	*β*	*γ*	*μ*_2_	*μ*_r2_	Repose Angle (◦)
1	1	1	−1	1	1	1	−1	−1	34.04
2	−1	1	1	−1	1	1	1	−1	34.35
3	1	−1	1	1	−1	1	1	1	37.60
4	−1	1	−1	1	1	−1	1	1	30.59
5	−1	−1	1	−1	1	1	−1	1	35.02
6	−1	−1	−1	1	−1	1	1	−1	33.63
7	1	−1	−1	−1	1	−1	1	1	27.52
8	1	1	−1	−1	−1	1	−1	1	30.68
9	1	1	1	−1	−1	−1	1	−1	33.13
10	−1	1	1	1	−1	−1	−1	1	35.62
11	1	−1	1	1	1	−1	−1	−1	35.74
12	−1	−1	−1	−1	−1	−1	−1	−1	27.25
13	0	0	0	0	0	0	0	0	32.99

**Table 5:** Variance analysis of Plackett–Burman test results.

Sources	Sum of Squares	*df*	Mean Square	*F*-Value	*p*-Value	Significance Ranking
Model	116.11	8	14.51	39.06	0.006 **	
*G*-*G*	0.42	1	0.42	1.12	0.3678	4
*ν*-*ν*	0.23	1	0.227	0.61	0.4915	5
*μ*_1_-*μ*_1_	64.27	1	64.27	172.96	0.0009 **	1
*μ*_r1_-*μ*_r1_	30.90	1	30.90	83.15	0.0028 **	2
*β*-*β*	0.04	1	0.04	0.10	0.7773	8
*γ*-*γ*	19.96	1	19.96	53.70	0.0052 **	3
*μ*_2_-*μ*_2_	0.19	1	0.19	0.52	0.5216	6
*μ*_r2_-*μ*_r2_	0.10	1	0.10	0.28	0.6321	7

Note: *df* is the abbreviation for degree of freedom, ** means this item is extremely significant (*p* < 0.01). Same notation used below.

**Table 6:** Design and results of the steepest ascent test.

Number	*μ*_1_	*μ*_r1_	*γ*	Repose Angle (◦)	Relative Error (%)
1	0.4	0.30	0.04	30.07	15.68
2	0.5	0.35	0.08	31.99	10.29
3	0.6	0.40	0.12	33.65	5.64
4	0.7	0.45	0.16	36.48	2.30
5	0.8	0.50	0.20	38.58	8.20
6	0.9	0.55	0.24	40.28	12.97

**Table 7:** Design and results of Box–Behnken test.

Number	*μ*_1_	*μ*_r1_	*γ*	Repose Angle (◦)
1	−1(0.6)	−1(0.4)	0(0.16)	33.89
2	1(0.8)	−1	0	36.91
3	−1	1(0.5)	0	35.20
4	1	1	0	38.26
5	−1	0(0.45)	−1(0.12)	34.17
6	1	0	−1	37.39
7	−1	0	1(0.20)	35.86
8	1	0	1	37.79
9	0(0.7)	−1	−1	34.86
10	0	1	−1	37.29
11	0	−1	1	36.38
12	0	1	1	37.46
13	0	0	0	36.70
14	0	0	0	36.54
15	0	0	0	36.61

**Table 8:** Variance analysis of regression model of Box–Behnken test.

Sources	Sum of Squares	*df*	Mean Square	*F*-Value	*p*-Value
Model	23.86	9	2.65	57.48	0.0002 **
*μ*_1_	15.77	1	15.77	342.01	<0.0001 **
*μ*_r1_	4.78	1	4.78	103.72	0.0002 **
*γ*	1.79	1	1.79	38.81	0.0016 **
*μ*_1_ *μ*_r1_	0.0003	1	0.0003	0.0063	0.94
*μ*_1_ *γ*	0.4198	1	0.4198	9.1	0.0295 *
*μ*_r1_ *γ*	0.4591	1	0.4591	9.96	0.0252 *
*μ*_1_^2^	0.5103	1	0.5103	11.07	0.0209 *
*μ*_r1_^2^	0.1191	1	0.1191	2.58	0.1689
*γ^2^*	0.0129	1	0.0129	0.2792	0.6198
Residual	0.2306	5	0.0461		
Lack of Fit	0.2191	3	0.073	12.67	0.074
Pure Error	0.0115	2	0.0058		
Cor Total	24.09	14			

Note: ** means this item is extremely significant (*p* < 0.01), * means this item is significant, (*p* < 0.05).

## Data Availability

The data presented in this study are available upon request from the corresponding author. The data are not publicly available due to the confidentiality of the subject research.
